# Effect of Traffic Noise and Relaxations Sounds on Pedestrian Walking Speed

**DOI:** 10.3390/ijerph15040752

**Published:** 2018-04-14

**Authors:** Marek Franěk, Lukáš Režný, Denis Šefara, Jiří Cabal

**Affiliations:** Faculty of Informatics and Management, University of Hradec Králové, Rokitanského 62, 500 03 Hradec Králové, Czech Republic; lukas.rezny@uhk.cz (L.R.); denis.sefara@uhk.cz (D.Š.); jiri.cabal@uhk.cz (J.C.)

**Keywords:** noise exposure, walking speed, stress, relaxation, urban nature

## Abstract

Exposure to noise in everyday urban life is considered to be an environmental stressor. A specific outcome of reactions to environmental stress is a fast pace of life that also includes a faster pedestrian walking speed. The present study examined the effect of listening to annoying acoustical stimuli (traffic noise) compared with relaxation sounds (forest birdsong) on walking speed in a real outdoor urban environment. The participants (*N* = 83) walked along an urban route of 1.8 km. They listened to either traffic noise or forest birdsong, or they walked without listening to any acoustical stimuli in the control condition. The results showed that participants listening to traffic noise walked significantly faster on the route than both the participants listening to forest birdsong sounds and the participants in the control condition. Participants who listened to forest birdsong walked slightly slower than those under control conditions; however, this difference was not significant. Analysis of the walk experience showed that participants who listened to forest birdsong during the walk liked the route more than those who listened to traffic sounds. The study demonstrated that exposure to traffic noise led to an immediate increase in walking speed. It was also shown that exposure to noise may influence participants’ perception of an environment. The same environment may be more liked in the absence of noise or in the presence of relaxation sounds. The study also documented the positive effect of listening to various kinds of relaxation sounds while walking in an outdoor environment with traffic noise.

## 1. Introduction

The negative health consequences of noise exposure have been studied frequently [1,2,3,4,5]. The non-auditory effects of noise on humans have been intensively studied in the last four decades. These effects include noise annoyance, and are linked with various psychological symptoms (headaches, argumentativeness, changes in mood and anxiety). They also impair cognitive performance. Moreover, chronic exposure to noise can cause sleep disturbance or cardiovascular diseases (e.g., Basner et al. [6]. Seidman and Standring [7], Stansfeld and Matheson [8]). Exposure to noise in everyday urban life is considered an environmental stressor [9]. One specific reaction to environmental stress is a fast pace of life, which was defined several decades ago by Werner, Altman, and Oxley ([10], p. 14) to be the “relative rapidity or density of experiences, meanings, perceptions and activities”. Researchers have analyzed the temporal aspects of various urban behaviors and have calculated a value that expresses an overall pace of life. A fast pace of life also includes a faster pedestrian walking speed, which may be a response to stimulatory overload and various urban stressors, including crowding and traffic noise [11]. Some studies showed that people walk faster in large cities compared with smaller towns [12]. A more detailed analysis showed that people tend to walk faster in urban streets with dense traffic and traffic noise [13] compared with calmer streets. This suggests that the fast pedestrian walking pace may be a spontaneous reaction to traffic noise. The present study examined the effect on walking speed of listening to annoying acoustical stimuli (traffic noise) compared to relaxation sounds (forest birdsong) in a real outdoor environment.

Investigations of pedestrian walking speed documented the phenomenon of a fast pedestrian speed in main downtown areas, as well as the negative health consequences of the fast pace of life in large cities. A pioneering study by Bornstein and Bornstein [11] reported high positive correlations between the walking speed of pedestrians and the size of the city. This finding repeatedly was supported in subsequent studies [14,15]. There is also evidence that the faster pace of life in large cities is associated with a greater likelihood of heart attacks (e.g., Levine and Norenzayan [12], Levine and Bartlett [16]). Levine, Lynch, and Lucia [17] interpreted movement speed and the speed of other daily activities as being parallel to Type A behavior patterns (which are a potential risk factor for heart disease) and even suggested using the term “Type A city”. More recently, Wiseman [18] compared the walking speeds of the inhabitants of 32 capital cities. Surprisingly, the walking speed in large cities increased by approximately 10% when compared with previous data found by Levine and Norenzayan [12] in the early 1990s. Thus, a fast pace of life in today’s cities, one of whose symptoms is a faster walking speed, could represent a potential risk factor that may negatively affect the well-being and health of city dwellers.

Although some authors suggested (e.g., Bornstein and Bornstein [11]) that traffic noise may be one factor that influences walking speed, this proposition has not been tested adequately. In our previous studies, we examined the effects of the visual and acoustical environmental features of surrounding environments on walking speed [13,19,20]. It was observed that participants walked faster in areas without urban greenery and with more traffic, higher noise, and more people than in areas with urban greenery and with less traffic, less noise, and fewer people. However, the effect of traffic noise was based only on subjective estimations of the acoustical characteristics of particular locations. Recently, Maculewicz, Erkut, and Serafin [21] experimentally examined how sound characteristics for specific environments affect walking pace. The participants listened to sounds of the seashore, a busy street, a restaurant, and busy offices, and simultaneously walked at their own preferred pace on an aerobic stepper. Their results indicated that the sounds of the seashore and a restaurant provoked a significantly slower pace than the sounds of streets and offices. The study documented not only the effect of traffic noise on walking pace, but also showed that listening to nature sounds may result in a decrease in walking speed.

Although traffic noise may cause perceived stress, there are opposite studies that show the restorative effects of urban nature. Some research demonstrates that living in areas with large amounts of urban greenery or only exposing individuals to a natural environment either in a visual or acoustic form results in decreased stress. It is known that residents of neighborhoods with a greater percentage of greenery have lower chronic stress [22,23,24]. Stress recovery, as measured through a variety of physiologic measures, was more rapid in the group that viewed natural scenes compared with a group that viewed urban scenes [25]. A large number of studies have documented that viewing surrogate nature (photographs, slides, paintings, window views, videos, and virtual computer-generated nature scenes) resulted in decreased stress, increased positive emotions, and decreased negative emotions [25,26,27,28,29,30,31,32]. In accordance with these findings, our recent study showed that the presentation of photographs of nature scenes prior to an outdoor walk decreased walking speed when contrasted with priming with photographs of shopping malls, and a control condition without any priming [33].

Although a large amount of studies examined the positive effect of viewing nature scenes, the effect of natural sound has been explored less substantially in experimental settings. It was confirmed that natural sounds tend to be evaluated as pleasant and support recovery, while technological sounds tend to be experienced as disturbing [34]. The exposure of natural sounds led to greater mood recovery after the presentation of annoying stimuli compared with human-caused sounds [35,36,37,38].

A further question regards the interaction between the effects of greenery and environmental sounds. The results of several studies suggested that visions of nature from a window or easy access to nearby green areas may reduce the negative impact of traffic noises; these might make the sound seem less annoying [39,40]. Interestingly, Lee, Hong, and Jeon [41] showed that the noise from a high-speed train was estimated as less annoying if the sound was presented with a picture containing a higher percentage of natural features. Viollon, Lavandier, and Drake [42] reported that birdsong and traffic noise were judged significantly more negatively where they were presented together with more urban visual scenes.

Moreover, congruency between a specific environment and sound also may play a role because people expect the appearance of specific sounds that are congruent with the physical features of the surrounding environment [43]. Brambilla and Maffei [44] demonstrated that the level of annoyance is lower and acceptability is higher when the sound is more congruent with the listener’s expectation. Jahncke, Eriksson, and Naula [45] examined the combined effect of diverse acoustical stimuli (nature sounds, quiet broadband noise, and office noise) and visual settings (office and an urban nature environment) on perceived restoration. They found that a picture of nature was more sensitive to the influence of auditory stimuli than an office picture.

The present study continues our previous investigations of pedestrian walking pace in a real outdoor urban environment [13,19,20,33]. To systematically examine the effects of different environmental sounds on walking speed, we asked participants to listen either to traffic noise or relaxation nature sounds while walking on an outdoor route. As previously demonstrated, certain features of the physical environment can also influence walking speed, such as the presence or absence of urban greenery. People tend to walk slower in an environment with higher perceived natural characteristics [19]. It is yet to be determined whether particular environmental sounds (traffic noise versus forest birdsong) may have the same effect on walking speed in different environmental settings, specifically on streets with traffic noise and a small amount of greenery or on a route with greenery without any noise.

The aim of the present study is to investigate the effect of listening to diverse environmental sounds on walking speed while walking in a real outdoor environment. It is supposed that listening to traffic noise increases walking speed, while listening to nature sounds decreases walking speed. Furthermore, the interaction between the effect of environmental features and sound will be examined.

## 2. Materials and Methods

### 2.1. Ethics Statement

Ethical approval (1/2017 FIM KM) was obtained from the Department of Management at the University of Hradec Králové for the experiment. All of the participants provided written informed consent. The participants signed a consent declaration in which they declared that they voluntarily agreed to participate in the experiment, and that they were informed about the experimental procedure. They could decide to stop being a part of the research study at any time without explanation. There were no known risks for participants in this study. The collected data will be anonymized, and used for the research purposes only.

### 2.2. Participants

Eighty-three undergraduates participated in the study. The students were young adults aged from 19 to 25 years (M age = 21.36 years, SD = 1.48); 48 were men and 35 were women. They were recruited from a range of fields of study (informatics, financial management, and tourism) at the University of Hradec Králové. They were compensated by partial course credit. Participants walked under three conditions: forest birdsong, traffic noise, and a control condition without hearing any (surrogate) sound. Each participant did only one condition.

### 2.3. Stimulus Material

There were two conditions with diverse sounds; the participants did not listen to any sound under the control condition. A track with relaxation sounds or a track with traffic noise was selected as the acoustical stimuli. The soundtrack from the video “Forest Birdsong—Relaxing Nature Sounds—Birds Chirping”, which is available on YouTube (https://www.youtube.com/watch?v=Qm846KdZN_c), was selected as the relaxation sound. The track consists of the sound of birds singing (Nightingale, Blackbird, Chaffinch, Cuckoo, and others), and a calm forest river. The soundtrack from the video “Hectic Kolkata (Calcutta)—India”, which is also available on YouTube (https://www.youtube.com/watch?v=IFc2KhKLiho), was selected as the traffic noise. The track contains traffic noise and noise from motorized vehicles, engine sounds, intense automobile horns, and human voices. The second track had to be modified because of its short length (9 min and 40 s), which did not correspond with the length of the participants’ walk. The track was modified using the software Audacity to have the length of 38 min by repeating it four times in succession. The sound levels of the tracks were adjusted to a comfortable level, and did not differ between participants. The mean sound pressure level was: crowded city noise—53 dBA, forest birdsong—56 dBA (the magnitude of the mean sound pressure level reflects the higher differences between the sound levels of a calm river and the birdsong). Participants listened to the tracks, which were played on a Nokia Lumia 520 (Nokia, Helsinky, Finland) with a Windows Phone 8.1 operating system, using the Nokia Music application, version 3.10.822.0 (Nokia, Helsinky, Finland).

The sounds were listened to through lightweight Genius HS-M200C headphones (KYE Systems Corporation, New Taipei City, Taiwan). For safety reasons, the headphones did not entirely mask sounds from the outside. Participants assigned to the control condition did not wear headphones while walking. Traffic noise in sections 1–4 of the walking route (Table 1) was about Lday = 55–60 dBA, and the traffic noise in sections 5–7 was about Lday = 50–55 dBA [46].

The participants were randomly assigned to a specific condition. The experiment was divided into one-hour blocks. Gender and the condition were balanced over each block in order to prevent effects of immediate changes of atmospheric conditions or traffic density. There were 17 males and 14 females in the forest birdsong condition. In the traffic noise condition, there were 14 males and 12 females, and in the control condition, there were 17 males and 9 females.

### 2.4. Walking Route

The experiment was conducted in the central area of Hradec Králové. This city has approximately 100,000 inhabitants. The walking route was a circuit with a length of 1.8 km. The first part of the route was a street with driving cars; the second part was a dense oak alley that led out into a noisy street with traffic. The walking route was situated to the area, where there were very few pedestrians walking. Thus, the movement of other pedestrians did not significantly affect the walking speed of the participants in the experiment. We chose seven sections for the analysis of walking speed (Table 1). The sections were chosen to provide a direct route and avoid crossing crossroads or other obstacles. The participants first moved from the university building to the starting point, which was about 300 m from the building. When they reached the end of section 7, they returned back and went along the same route in the opposite direction.

### 2.5. Measurement of Walking Speed

The participants walked with a small video camera Sony Bloggie MHS-PM5K (Sony, Tokyo, Japan) on a belt around their waist (size 19 mm × 108 mm × 55 mm, weight 110 g). The environment, the participant’s feet, and the participant’s arms were captured through a fish eye lens. The beginning and end of each section of the route was indicated by a line that was drawn on the sidewalk. An evaluator marked two frames of the video recording to create the beginning and end of the annotation for each particular track section in the software Elan (Nijmegen, The Netherlands, see https://tla.mpi.nl/tools/tla-tools/elan/). Every annotation represented the entire section of the track, so that the extent of the time that the subjects spent there could be determined. This enabled the calculation of the average speed reached by the participants in all of the sections.

### 2.6. Evaluation of Walk Experience

The participants rated their experience during their walk and their enjoyment of the environment using the following five items: (1) I was fine during the walk, (2) It was a pleasant time, (3) I liked the route I went through, (4) While walking, I often observed the surroundings, and (5) The sounds I listened to from my headphones bothered me. They were required to rate their agreement or disagreement with these items using a seven-point Likert-type scale with anchors where 1 = absolutely disagree, and 7 = absolutely agree.

### 2.7. Procedure

The participants individually walked around the route. They were sent successively to the route in periods of five minutes. Participants were instructed to walk through the route according to their normal walking speed. We used the description “normal” to discourage participants from walking as fast as possible to pass the route in the shortest possible time or, on the other hand, to move too slowly, such as walking for restorative purposes. Further, they were asked to not stop walking, and not call or speak with other people. The walking route was marked by orange arrows painted on the surface of the sidewalk. Participants were asked to complete a questionnaire describing their evaluation of the walk experience after the walk. Participants were not informed about the goal of the study. Before the experiment began, the participants were questioned as to whether they suffered from any current stress or anxiety (an exam, etc.). All of them gave a negative response.

The study was conducted in 2017 on three weekdays: May 2, May 3, and May 4. On the morning of May 2, it was sunny and the temperature was about 8 ℃. On the afternoon of May 2, it was cloudy and the temperature was about 14 ℃. On the morning of May 3, it was sunny and the temperature was about 11 ℃. On the afternoon of May 3, it was cloudy and the temperature was about 15 ℃. On the morning of May 4, it was sunny and the temperature was about 12 ℃. On the afternoon of May 4, it was cloudy and the temperature was about 15 ℃. The grass and trees along the route were green.

### 2.8. Data Analysis

**Figure 1:**
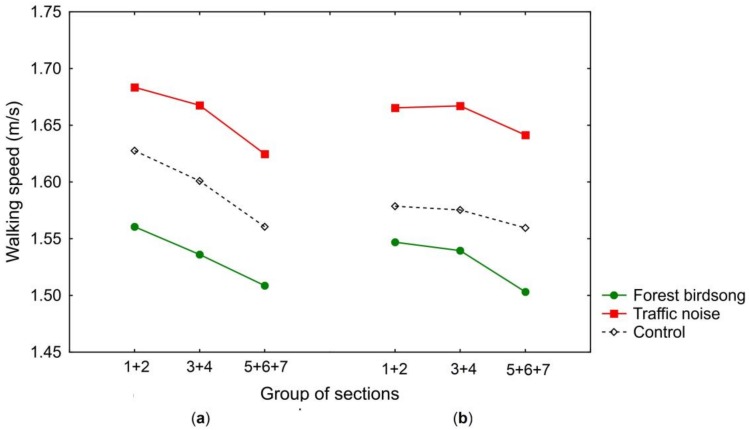
Average walking speeds (m/s) in particular groups of sections of the route. (**a**) The direction of the walk from section 1 to section 7. (**b**) The direction of the walk from section 7 to section 1.

The mean walking speeds were calculated for specific sections of the route. A three-way mixed analysis of variance (ANOVA) was conducted to analyze the effects of acoustic conditions (forest birdsong, traffic noise, or control condition), the route’s environmental properties (the section of the route), and the direction of the walk (from section 1 to section 7 or from section 7 to section 1) on the walking speeds in the specific sections. The acoustic condition was selected as the between-subjects factor, while the section of the route and the direction of the walk were selected as the within-subjects factors. The walking speed was selected as the dependent variable. The data were checked for sphericity by Mauchly’s test of sphericity. If the assumption of sphericity was violated, a Greenhouse-Geisser correction was applied based on the test’s Epsilon (ε). Since some sections on the walking route had similar environmental features, we joined similar sections into three groups (i.e., sections 1 + 2 = group 1, sections 3 + 4 = group 2, sections 5 + 6 + 7 = group 3). The score for each group was the mean across included sections (Figure 1). Differences between reported evaluations of the walk experience under particular conditions were compared by using a one-way ANOVA or *t*-test for independent samples. Statistical analyses were conducted using the Statistica 12 software (Stat Soft, Inc., Palo Alto, CA, USA).

## 3. Results

### 3.1. Analysis of Walking Speed

The results revealed an overall faster walking speed under the traffic noise condition (mean = 1.65 m/s, SD = 0.11), and a slower walking speed under the control condition (mean = 1.58 m/s, SD = 0.13); the slowest walking speed was under forest birdsong sounds (mean = 1.53 m/s, SD = 0.12). The average walking speeds in the particular sections of the route are shown in Table 2.

A three-way mixed ANOVA was conducted to access the effects of the condition (forest birdsong, traffic noise, control condition), the direction of the walk on the route (direction 1: from section 1 to section 7; direction 2: from section 7 to section 1), and the section of the route (group 1 = sections 1 + 2, group 2 = sections 3 + 4, group 3 = sections 5 + 6 + 7) on walking speed. There was homogeneity of variances, as assessed by Levene's test for equality of variances (*p* > 0.05). A Greenhouse-Geisser correction was applied where the assumption of sphericity was violated as assessed by Mauchly’s test of sphericity. A statistical significance of a simple two-way interaction and a simple main effect was accepted at a Bonferroni-adjusted alpha level of 0.016.

The ANOVA indicated a statistically significant between-subjects main effect of the condition (*F*_2, 80_ = 7.759, *p* < 0.001, η^2^ = 0.162). A post hoc analysis with a Bonferroni adjustment showed that: the participants listening to traffic noise walked significantly faster on the route than participants listening to forest birdsong sounds (*p* = < 0.001), the participants listening to traffic noise had a tendency to walk faster than participants in the control condition (*p* = 0.085), and the participants in the control condition walked non-significantly faster than participants listening to forest birdsong sounds (*p* = 0.337).

The ANOVA indicated a statistically significant within-subjects main effect of the group of sections (*F*_1.593, 127.480_ = 79.550, *p* < 0.001, η^2^ = 0.499, ε = 0.797). A post hoc analysis with a Bonferroni adjustment showed that the participants walked significantly faster in the group of sections 1 + 2 than in the group of sections 3 + 4 (*p* < 0.001), significantly faster in the group of sections 3 + 4 than in the group of sections 5 + 6 + 7 (*p* < 0.001), and also significantly faster in the group of sections 1 + 2 than in the group of sections 5 + 6 + 7 (*p* < 0.001).

The ANOVA also indicated a non-significant within-subjects main effect of the direction of the walk (*F*_1, 80_ = 2.973, *p* = 0.089, η^2^ = 0.036). The significant main effect of the direction of the walk appeared only under the control condition, when participants walked significantly faster in direction 1 (*p* < 0.05).

Since the ANOVA revealed a statistically significant three-way interaction between the sections that were walked, the direction of the walk, and the condition (*F*_3.253, 130.103_ = 2.942, *p* = < 0.05, η^2^ = 0.069, ε = 0.813), a further analysis was conducted. A statistical significance of a simple two-way interaction and a simple main effect was accepted at a Bonferroni-adjusted alpha level of 0.016. There was a statistically significant simple two-way interaction between the section walked and the direction of the walk under the condition traffic noise (*F*_1.580, 39.505_ = 5.559, *p* < 0.05, η^2^ = 0.182, ε = 0.790), and between the section walked and the direction of the walk under the control condition (*F*_2, 50_ = 13.979, *p* < 0.001, η^2^ = 0.359), but not under the forest birdsong condition (*F*_1.493, 44.79_ = 1.734, *p* = 0.185, η^2^ = 0.055, ε = 0.747). For the forest birdsong condition, a post hoc analysis with a Bonferroni adjustment confirmed statistically significant differences between pairwise comparisons of all of the sections (*p* < 0.001). The data also indicated the non-significant effect of the direction of the walk (*p* = 0.630) under the forest birdsong condition. A detailed analysis of the significant two-way interaction showed that there was a statistically significant simple main effect of the section walked for the traffic noise condition in the direction of walk 1 (*F*_2, 50_ = 21.233, *p* < 0.001), for the condition traffic noise in the direction of walk 2 (*F*_1.539, 38.467_ = 9.697, *p* <.001, ε = 0.769), for the control condition in the direction of walk 1 (*F*_1.505, 37.633_ = 25.690, *p* < 0.001, ε = 0.753), but not for the control condition in the direction of walk 2 (*F*_1.405, 35.114_ = 4.320, *p* < 0.05, ε = 0.702). For the condition traffic noise in the direction of walk 1, a post hoc analysis with a Bonferroni adjustment showed a statistically significant difference between walking speeds in the groups of sections 1 + 2 and 5 + 6 + 7, between walking speeds in the group of sections 3 + 4, but not between walking speeds in the groups of sections 1 + 2 and 3 + 4. For the traffic noise condition in the direction of the walk 2, there was a statistically significant difference between walking speeds in the groups of sections 3 + 4 and 5 + 6 + 7, but not between walking speeds in the groups of sections 1 + 2 and 5 + 6 + 7, nor between walking speeds in the groups of sections 1 + 2 and 3 + 4. For the control condition in the direction of the walk 1, a post hoc analysis with a Bonferroni adjustment confirmed statistically significant differences between pairwise comparisons of all of the group sections (*p* < 0.001). To sum, the statistical analyses revealed that listening to traffic noise resulted in a significantly faster walking speed than listening to forest birdsongs, and almost a significantly faster speed than in the control condition. However, although listening to forest birdsong resulted in a slower walking speed in contrast to the walking speed in the control condition, this difference was not significant. The experimental condition also influenced differences in walking speeds in particular sections of the route. Finally, the section of the route significantly affected the overall walking speed; participants achieved the fastest walking speed in the group of sections 1 + 2, followed by the group of sections 3 + 4 and the group of sections 5 + 6 + 7.

### 3.2. Evaluation of Walk Experience

The scores for particular items are in Table 3. It was examined how the agreement with the statement “I was fine during the walk” was related to the type of acoustic stimulus to which participants listened. One-way ANOVA indicated a statistically significant effect of the type of condition (*F*_2, 80_ = 3.986, *p* < 0.001, η^2^ = 0.17). A post hoc Tukey test indicated significant differences between the forest birdsong and traffic noise conditions, and between traffic noise and the control condition. The participants listening to forest birdsong sounds felt more fine during the walk than those listening to traffic noise. The participants listening to traffic noise felt less fine during the walk than those under the control condition.

One-way ANOVA indicated that agreement with the statement “It was a pleasant time” was statistically significantly influenced by the type of acoustic stimulus (*F*_2, 80_ = 11.273, *p* < 0.001, η^2^ = 0.22). A post hoc Tukey test indicated significant differences between the conditions of forest birdsong and traffic noise, and between traffic noise and the control condition. The walk was a more pleasant experience for participants listening to forest birdsong sounds than for the participants listening to traffic noise. The walk was a less pleasant experience for the participants listening to traffic noise than for those under control conditions.

One-way ANOVA indicated that agreement with the statement “I liked the route I went through” was statistically significantly influenced by the type of acoustic stimulus (*F*_2, 80_ = 4.705, *p* < 0.05, η^2^ = 0.11). A post hoc Tukey test indicated significant differences between the forest birdsong and traffic noise conditions. Participants listening to forest birdsong liked the route more than those listening to traffic noise. However, the type of acoustic stimulus did not significantly influence agreement with the statement “While walking, I often observed the surroundings.” (*F*_2, 80_ = 1.295, *p* = 0.280).

A *T*-test for independent samples indicated significant differences between the level of agreement with the statement “The sounds I listened to from my headphones bother me” in both conditions with acoustic stimuli. The traffic noise bothered participants more than forest birdsong sounds (*t* = 4.077, *p* < 0.001, Cohen’s *d* = 1.15).

## 4. Discussion

This study examined the effects of listening to diverse environmental sounds on walking speed while walking in a real outdoor environment. As expected, the results showed that listening to traffic noise increased participants’ walking speed on the urban route. In contrast, listening to the relaxing sounds of forest birdsong made their walking speed slightly slower.

Verbal descriptions of participants’ walking experiences revealed negative evaluations of various aspects of the walk while listening to traffic noise. Listening to traffic noise was annoying; participants who listened to traffic noise estimated their walk to be less pleasant, and they liked the route less than the participants who listened to relaxation sounds or the participants under control conditions. This further supports the existence of an association between negative reactions to traffic noise and a faster walking speed.

In contrast to traffic noise, participants listening to relaxation sounds liked the route more and evaluated their walk as more pleasant than those who were under a traffic noise condition or under control conditions (although statistically non-significantly). In addition to previous studies that reported that visual natural stimuli may make traffic noise seem less annoying [39,40], we observed that acoustic natural stimuli resulted in a higher level of liking the visual properties of the environment when compared to acoustic stimulation with traffic noise.

In the introduction, we discussed the long-term non-auditory effects of noise exposure. Unfortunately, the pace of life is an insufficiently explored concept that has been investigated independently from this research area. There is still a lack of experimental evidence regarding which environmental variables could influence the pace of life, and whether particular components of the pace of life could interact with one another. Nevertheless, there was agreement that the increased tempo of the pace of life is also manifested by the increased walking speed of urban dwellers [12,17]. Our research attempted to link these two research areas. In accord with the recent study by Maculewicz et al. [21] that was conducted in a laboratory, the present experiment performed in a real outdoor environment revealed that participants tend to react to listening to a soundtrack of a traffic noise through walking faster in contrast to listening to natural sounds. In addition, these findings are supported by our previous research, where we found that people walk slightly faster in areas with traffic noise than they do in areas without traffic [13,19]. Importantly, in the present experiment, the traffic noise has not been presented to the participants as a loud unpleasant sound; it was adjusted to a comfortable level. Thus, a possible explanation of the observed effect is that participants might be influenced by listening to a specific soundscape that is associated with rapidity and density, rather than by a proper effect of intense sounds. Clearly, further research is needed to explore the effect of diverse acoustical stimuli at various sound levels.

Further, we analyzed the interactions between the effects of a specific environment and the presented sounds. In our previous studies of walking speed, which were conducted on the same route [19] without listening to any acoustic stimuli, we observed the slowest walking speed in sections 5–7, a faster walking speed in sections 3–4, and the fastest walking speed in sections 1–2. Roughly identical patterns of walking speed in the particular sections were found in the present experiment under the condition with traffic noise in both directions of the walk. As expected, listening to the traffic noise accelerated the walking speed in contrast to the other conditions, but visual properties and the high natural character of sections 5–7 (the dense oak alley) resulted in a deceleration of the walking speed in this area. In accord with findings by Jahncke, Eriksson, and Naula [45], who reported that a picture of nature was more sensitive to the influence of auditory stimuli than an office picture, one could suppose that a strong feeling of incongruency between listening to traffic noise and viewing a calm natural environment might result in a faster walking speed in this area. However, we observed the opposite tendency, which speaks more for the strong positive effect of the natural environment than for the effect of incongruency between visual and acoustic properties of the environment. Thus, in an accord with previous studies [39,40], we can speculate that visions of a natural environment reduced the negative impact of the traffic noise in this section of the route. However, we do not have empirical evidence for this claim, because we did not directly perform a subjective evaluation of the walking experience on the route.

In contrast, there were no significant differences in participants’ walking speeds between the particular sections under the forest birdsong condition. Although the data revealed a slight deceleration of walking speed in sections 5–7, which had a high natural character, these differences were not significant. It seems that the acoustic stimuli that contained sounds from the natural environment had a stronger effect than the visual properties of the route, and affected the participants over the course of the whole experiment. Moreover, the whole walking route contained some level of greenery; thus, participants probably did not feel incongruency between the sounds and visual properties of the route anywhere.

Finally, although we found significant differences between walking speeds in particular sections in the direction of the walk that led from section 1 to section 7 in the control condition, in the backward direction of the walk, the differences were not significant. Moreover, the participants walked more slowly from section 1 to section 7 than they did in the opposite direction. A similar tendency was observed in the condition with traffic noise in sections 5–7. It is supposed that this behavior reflects some form of habituation, which is perhaps related to familiarity with the route and its environmental properties.

It is also worth commenting on the selection of acoustical stimuli in the present study. The problem is that isolated environmental sounds probably do not offer a similar amount of unambiguous information about an environment as visual stimuli or combination of acoustic and visual stimuli do. We used birdsong as the stimuli in our experiment, because it was found that this type of natural sound is most commonly associated with perceived stress recovery and attention restoration [47]; it is also associated with green spaces, as well as spring and summer [48]. The natural environment soundscape is more complex; it may contain wind blowing and rustling leaves, etc. The problem is that those sounds may not be easily separated from some technological sounds. For instance, Haga et al. [49] showed that participants perceived an ambiguous sound consisting of pink noise with white noise interspersed either as a natural sound (waterfall) or as an industrial sound in accordance with instructions given prior to the experiment. The appropriate selection of a traffic noise faced a similar problem. An acoustical stimulus may be ambiguous when there is no visual information accompanying it. Therefore, we decided to use sounds consisting of very hectic crowded city noise in order for them to be unambiguously identified as a traffic noise. If the soundtrack would have been a classical traffic noise, it is possible that we could have registered a weaker effect. On the other hand, the sound level of this crowded city noise was adjusted to a comfortable level, which somewhat reduced its effect.

The study has some limitations. First, the headphones did not entirely mask outdoor sounds under the nature sounds condition, due to safety reasons. Thus, participants also slightly heard noise from outside the experiment. Although this arrangement may reflect real situations, when people are walking outdoors and simultaneously listening to music or relaxation sounds from headphones, it did not entirely change the soundscape of the environment. Second, although there was car traffic in sections 1, 2, 3, and 4 of our walking route, the street was not a typical example of a busy urban highway. Thus, it is possible that phenomena associated with the observed effects of congruency/incongruency between the environment and sound [43,44] would be less pronounced.

Further, although we supposed that the participants in the study had good and symmetrical hearing without any hearing impairments or asymmetric hearing loss, we did not measure participants’ hearing.

It is worth mentioning that the walking speed that was observed in the present experiment was relatively fast in all of the experimental conditions. It should be pointed out that our participants were young healthy adults, who were mostly involved in various sports. Moreover, there were not any obstacles, nor a flow of other pedestrians on the walking route. In comparison, Bohannon [50] asked his participants (adults aged 20–79 years) to walk at a comfortable pace or at maximum gait speed. He reported an average comfortable gait speed about 1.40 m/s and a maximum gait speed about 2.50 m/s for males and females in their twenties. Interestingly, no one examined the walking speed of various social groups, specifically the walking speed of young university students. Consistent with the present study, we observed similarly fast walking speeds in our previous experiments that were conducted with university students on routes in the same city [13,19,20,33], although different paradigms and methodologies of measurement have been used.

The present experiment, which was conducted in a real outdoor environment, has greater ecological validity. However, the drawback of the methodology used is that it was not possible to control all of the external variables, such as immediate changes in atmospheric conditions, weather, or traffic density. Therefore, we tried to reduce the impacts of these variables by balancing the conditions during the days on which the experiment was performed.

## 5. Conclusions

In conclusion, our study convincingly showed that exposure to traffic noise led to immediate increases in walking speed. Of course, a faster walking pace is not an undesirable behavior of urban pedestrians, such as if it is, for instance, a part of a sport or recreational activity. However, as previously demonstrated, a fast walking speed in the context of the overall fast pace of life as a response to stressful environmental stimuli may have negative health consequences. Moreover, it was also shown that exposure to noise may influence the perception of an environment. The same environment may be more liked in the absence of noise or in the presence of relaxation sounds. Finally, the study also documented the positive effect of listening to relaxation sounds while walking in an outdoor environment with traffic noise.

## Figures and Tables

**Table 1:** Walking route. The description of particular sections where walking speed was measured. For additional route details, see https://maps.google.com, location: Hradec Kralove, Czech Republic, *Orlicke nabrezi*.

Section	Length (m)	Environmental Layout	Street
1	60	grass, trees, buildings, traffic,	Brno Street—Technical High School
2	55	grass, trees, buildings, traffic	Brno Street—Business High School
3	100	grass, trees, traffic,	Brno Street—Business Center
4	100	grass, trees, traffic	Brno Street—Botanical Garden
5	60	dense oak alley, no traffic	Brno Street—Malšovická Street
6	75	dense oak alley, no traffic	Flošna—tree alley
7	90	dense oak alley, no traffic	Flošna—parking

**Table 2:** The average walking speeds (m/s) in specific sections of the route for the two experimental conditions (forest birdsong, traffic noise), and the control condition for both directions of the walk. Direction 1 is from section 1 to section 7, and direction 2 is from section 7 to section 1.

Section	Forest Birdsong		Traffic Noise		Control		Forest Birdsong		Traffic Noise		Control	
	Mean	SD	Mean	SD	Mean	SD	Mean	SD	Mean	SD	Mean	SD
	Direction 1						Direction 2					
1	1.56	0.13	1.68	0.12	1.63	0.15	1.55	0.12	1.66	0.11	1.57	0.11
2	1.56	0.13	1.68	0.12	1.63	0.15	1.55	0.13	1.67	0.11	1.58	0.12
3	1.54	0.12	1.67	0.12	1.60	0.15	1.55	0.13	1.67	0.13	1.58	0.13
4	1.54	0.13	1.67	0.13	1.60	0.15	1.53	0.13	1.67	0.11	1.57	0.13
5	1.52	0.13	1.63	0.12	1.58	0.15	1.51	0.12	1.66	0.12	1.57	0.13
6	1.51	0.12	1.63	0.12	1.55	0.15	1.50	0.12	1.64	0.12	1.56	0.12
7	1.50	0.12	1.61	0.13	1.54	0.15	1.49	0.13	1.63	0.12	1.54	0.13

**Table 3:** Evaluation of the walk experience. The level of agreement with particular items. The scale ranged from 1 to 7.

Item	Forest Birdsong		Traffic Noise		Control	
	Mean	SD	Mean	SD	Mean	SD
I was fine during the walk.	6.42	0.62	5.38	1.39	6.12	0.86
It was a pleasant time.	5.71	1.13	4.27	1.22	5.23	1.11
I liked the route I went through.	6.29	0.82	5.65	1.02	6.20	0.57
While walking, I often observed the surroundings.	6.16	1.19	5.65	1.44	5.77	1.14
The sounds I listened to from my headphones bother me	2.45	2.10	4.81	2.26	-	-

## Supplementary Materials

Supplementary File 1
